# Host-driven diversification of gall-inducing *Acacia *thrips and the aridification of Australia

**DOI:** 10.1186/1741-7007-5-3

**Published:** 2007-01-26

**Authors:** Michael J McLeish, Thomas W Chapman, Michael P Schwarz

**Affiliations:** 1 South African National Biodiversity Institute, Kirstenbosch Research Centre, Private Bag X7, Claremont, Cape Town, 7735, Republic of South Africa; 2 Department of Biology, Memorial University, St. John's, Newfoundland, A1B 3X9, Canada; 3 Flinders University, GPO Box 2100, Adelaide, South Australia, 5001

## Abstract

**Background:**

Insects that feed on plants contribute greatly to the generation of biodiversity. Hypotheses explaining rate increases in phytophagous insect diversification and mechanisms driving speciation in such specialists remain vexing despite considerable attention. The proliferation of plant-feeding insects and their hosts are expected to broadly parallel one another where climate change over geological timescales imposes consequences for the diversification of flora and fauna via habitat modification. This work uses a phylogenetic approach to investigate the premise that the aridification of Australia, and subsequent expansion and modification of arid-adapted host flora, has implications for the diversification of insects that specialise on them.

**Results:**

Likelihood ratio tests indicated the possibility of hard molecular polytomies within two co-radiating gall-inducing species complexes specialising on the same set of host species. Significant tree asymmetry is indicated at a branch adjacent to an inferred transition to a *Plurinerves *ancestral host species. Lineage by time diversification plots indicate gall-thrips that specialise on *Plurinerves *hosts differentially experienced an explosive period of speciation contemporaneous with climatic cycling during the Quaternary period. Chronological analyses indicated that the approximate age of origin of gall-inducing thrips on *Acacia *might be as recent as 10 million years ago during the Miocene, as truly arid landscapes first developed in Australia.

**Conclusion:**

Host-plant diversification and spatial heterogeneity of hosts have increased the potential for specialisation, resource partitioning, and unoccupied ecological niche availability for gall-thrips on Australian *Acacia*.

## Background

Determining the driving forces behind the diversification of phytophagous insects remains a central challenge to biology. Increases in insect species numbers can be expressed as an outcome of their feeding on plants [1,2]. The evolution of this type of ecological relationship between specialists and patterns of host-plant affiliation has been well documented [3-12]. The majority of phytophagous insect species are specifically associated with, and generally show preference for, single plant families, and this relationship is presumed to be evolutionarily conservative. This association is displayed in the narrow food specialisation and life habits of the majority of insect species. The mechanisms responsible for the generation of diversity in plant-insect systems are usually thought to result from cospeciation, or frequent host switches followed by specialisation. Host range expansion is believed to provide opportunities for vicariance, and the subsequent effects of selection or drift fuels the diversification of specialists [13]. This is in line with predictions that reciprocal radiations should occur for some insect-plant associations [4,14,15] where the proliferation and distribution of endoparasites and their hosts are expected to emulate one another.

The evolution of endemic biodiversity in contemporary Australia has primarily arisen over a period of increasing aridity during the late Tertiary period [16-20]. Truly arid landscapes appear some time towards the end of the Miocene, and more recently, dramatic changes to the terrestrial environment occurred during the Quaternary period, when glacial-interglacial cycling drove a rapid increase in desertification. The aridification of Australia modified habitat enhancing the evolution of flora, including sclerophylly. Range expansion and contraction of sclerophylly during glacial cycling [21-23] would be expected to provide opportunity for explosive speciation [15-17,24-31]. However, the proposal that faunal and floral diversification in Australia has been partly driven by climatic glacial-interglacial cycling effects over the last 2–3 million years remains a point of conjecture and is difficult to test [32].

Global patterns of gall-inducing species richness are believed to increase from mesic to xeric environments across transitional vegetation gradients from mesophytic to sclerophyllous plants [29,33-36]. Species-specific gall induction by thrips (Thysanoptera: Phlaeothripidae) on closely related desert *Acacia *(Leguminosae) trees has likely evolved as a consequence of the developing arid landscape [27,37-39]. *Acacia *is a prominent endemic Australian xeromorphic plant genus [39] and the most speciose plant genus on the continent, distributed throughout arid and semi-arid regions that cover over 70% of the Australian landmass [26,40]. The presence of sclerophyllous phyllodes (leaf-like modified petiole) is characteristic of *Acacia *on which gall-thrips induce galls [41] and are an adaptation to conserve water and to low nutrient environments [18,42-44]. *Acacia *population subdivision is partly determined by soil type mosaics [45-48] and this patchwork of nutrient poor sand dunes, sand plains and rocky ranges provided the sclerophylly habitat and opportunity for speciation [16,24,32]. Patchy distributions of *Acacia *are expected to contribute to heightened extinction and speciation rates [49]. This type of environmental heterogeneity can potentially also provide a foundation for increased specialisation and resource partitioning [50].

*Acacia *gall-thrips are a monophyletic group, distinct from other gall-inducing groups in Australia, implying that gall induction had a single origin in this case [51]. *Acacia *gall-thrips have likely undergone diversification as a result of cospeciation, highly restricted host-plant switching, or both processes [52,53]. The beginning of extensive aridification in Australia dates from the Late Miocene approximately 10–15 million years ago. The origin of *Acacia *gall-thrips likely arose in conjunction with the drying of Australia during this period [53]. In this study, we argue that host-specific gall-inducing thrips have diversified in close accordance with evolution of highly-related host *Acacia *in the subgenus *Phyllodineae *(DC.) Seringe, comprising more than 960 species largely confined to the Australian arid-zone [40]. Using a phylogenetic approach, we demonstrate that net increases in gall-thrips diversification are closely linked with host range expansion and aridification in Australia, particularly during the Quaternary period.

Gall-thrips diversity partly depends on a highly specialised affiliation with host *Acacia *species, where all described species are monophagous [53]. Galls are paramount in providing food, shelter, and a vessel for reproduction. The development and growth of galls has been widely correlated with the feeding activity and nutritional requirements of the galler [27]. Single females, or a female-male pair, induce a gall on the phyllode of a specific host plant and become fully enclosed within a few days after initiation. Extreme host specificity is evident in the morphologies of galls induced on different host species, and are invariant in appearance given any particular host species affiliation. Gall morphology amongst a species takes a generalised form of either an elongate or pouched structure, with more discrete characteristics evident for galls induced on specific hosts [28]. Elongate and pouched gall-types vary in appearance by the presence/absence of either spiky protrusions or prominent longitudinal ridges. Gall induction by founders is only permitted when new phyllode growth is available for inoculation. This event is usually dictated by local, infrequent, and unpredictable arid-zone rainfall patterns.

Sections *Plurinerves *and *Juliflorae *of the subgenus *Phyllodineae*, host to all but one gall-thrips species, do not appear to represent monophyletic groups but remain closely related to one another [54,55]. Species within these sections are believed to be relatively young compared to other sections in the subgenus *Phyllodineae *and more susceptible to speciation during the Pleistocene climatic cycling [54]. Semi-arid transitional climatic zones on the periphery of the interior arid zone have been proposed as having been particularly favourable for floral [21,31,32] and faunal [23,56,57] speciation. The lack of sequence divergence within the chloroplast genome of host *Acacia *and their close relatives, as well as evidence of a rapid morphological radiation among *Acacia *in subgenus *Phyllodineae*, is symptomatic of a relatively young group [54]. Speciation in the *Plurinerves *is believed to have occurred over a relatively short period as widespread aridity developed in the late Tertiary and early Quaternary periods [16,17] and that tropical section *Juliflorae *is more ancient than the *Plurinerves *section [58].

The addition of 29 undescribed taxa are added to all described *Acacia *gall-thrips species [51] to infer diversification hypotheses. These new taxa have previously been considered cryptic species (also referred to hereafter as morphotypes) and more recently believed to be groups of monophagous sister-species [52,53,59]. Recent work has raised one of these morphotypes to species status [59]. The extension of the gall-thrips phylogeny with the addition of these taxa is now believed to include a great majority of known types, although formal species delimitation is yet to be undertaken. We approached this study in two steps. First, to infer biogeographical processes acting on the genesis of diversity in gall-thrips/*Acacia *interactions, we made the assumption that gall-thrips diversification and host radiations were closely linked, and used species-level phylogenetic analyses and penalised likelihood approaches to date gall-thrips lineage diversification. Second, to address hypotheses of rate increase in gall-thrips diversification, expected as an outcome of *Acacia *radiations, we used likelihood ratio tests to identify late-branching molecular polytomies correlated with periods of explosive radiation, and tree asymmetry methods to identify rate changes.

## Results

### Phylogenetic analysis

**Figure 1:**
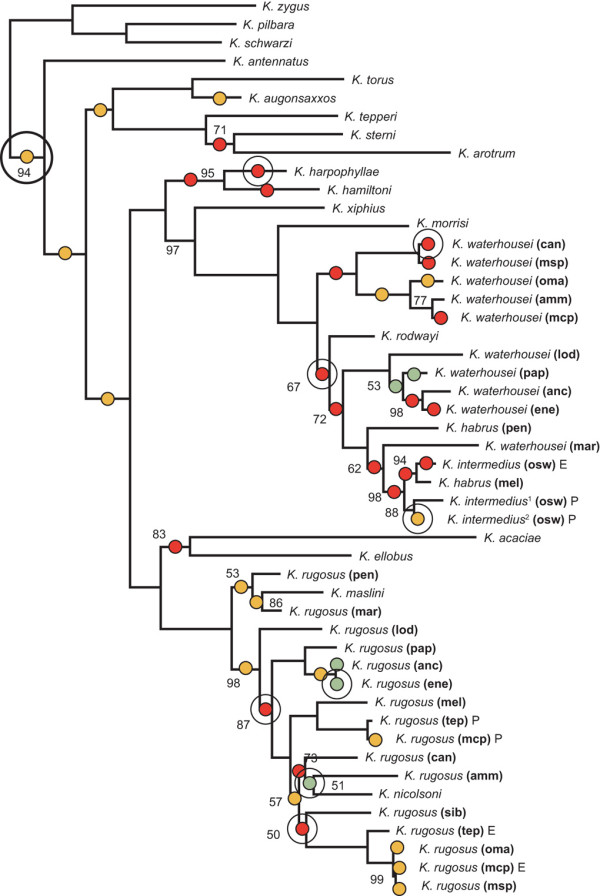
**Bayesian Consensus phylogram**. Posterior probabilities (100% probabilities not indicated) and mean branch lengths are derived from 3,000 trees taken from a sample of 5 million generations, sampling every 500^th ^generation. Six separately modeled partitions comprising 1st, 2nd, and 3rd *COI *codons and separate *EF-1α*, *wingless*, and *16S *sites were used to generate branch lengths not significantly different from zero derived from likelihood ratio tests of different gene partitions. Green dots indicate 6/6 partitions with branch lengths not significantly different from zero, red dots indicate 5/6 partitions, and yellow dots indicate 4/6 partitions. The intermediate sized open circles indicate branch lengths not significantly different from zero when the data was not partitioned. The large open circle shows the branch where a significant diversification rate change was detected. Abbreviations for *Acacia *host races are as follows: *A. ammophila *(amm), *A. ancistrophylla *(anc), *A. cana *(can), *A. enervia *(ene), *A. loderi *(lod), *A. maranoensis *(mar), *A. melvillei *(mel), *A. microcephala *(mcp), *A. microsperma *(msp), *A. omalophylla *(oma), *A. oswaldii *(osw), *A. papyrocarpa *(pap), *A. pendula *(pen) and *A. tephrina *(tep).

**Figure 2:**
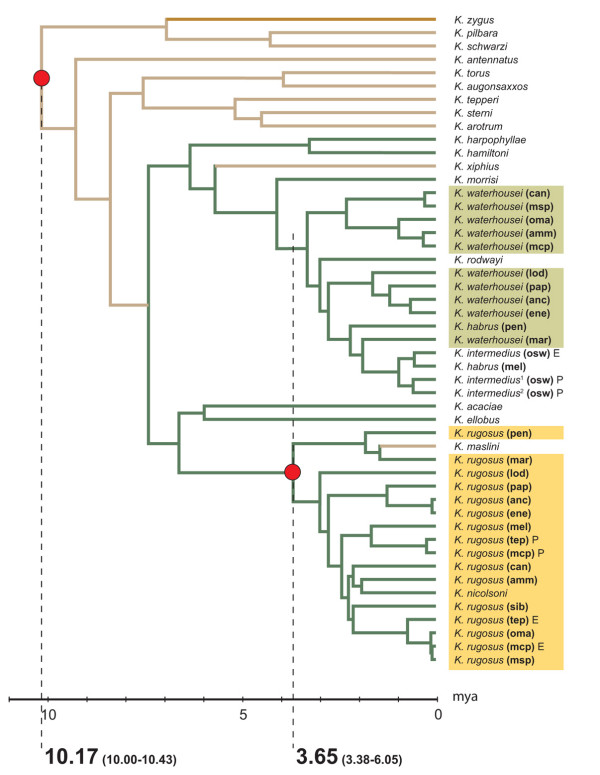
**A penalised likelihood chronogram of our Bayesian consensus tree**. The dark tan coloured branche indicates an ancestral affiliation with those of host section *Phyllodinae*; tan coloured branches indicate *Juliflorae *ancestral host affiliation; and green coloured branches indicate *Plurinerves *ancestral host affiliation. Red node circles show constrained calibration points between 10–15 mya and 2–5 mya. The coloured boxes indicate the *K. rugosus *and *K. waterhousei *species complexes. The time scale is in millions of years ago (mya).

**Figure 3:**
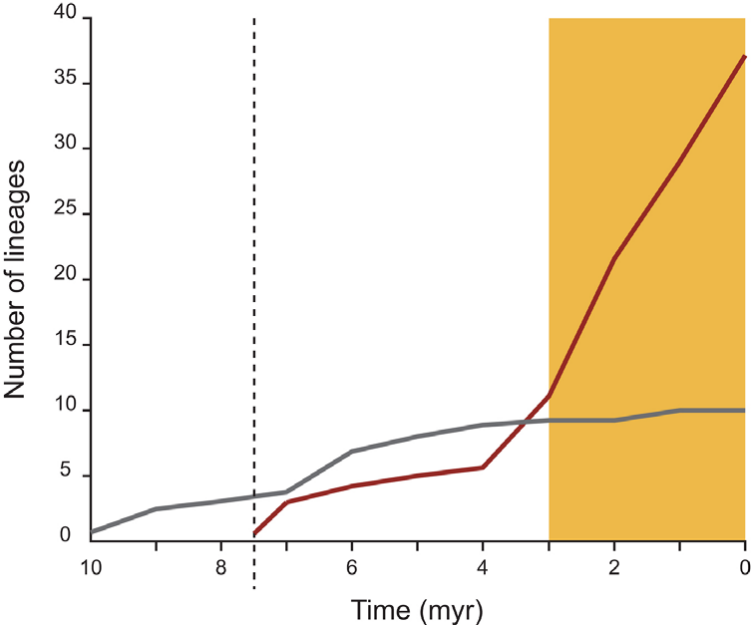
**Lineage by time diversification plot**. The plot shows differential rates of gall-thrips diversification that specialise on either *Plurinerves *(red line) or *Juliflorae *(grey line). The gold rectangle indicates the Quaternary period and a dotted line that indicates the inferred host switch to a *Plurinerves *ancestor. Lineage frequencies were counted using the Penalised Likelihood chronogram (Figure 2).

The Bayesian phylogenetic inference shows a high level of support for each of the clades containing the *Kladothrips rugosus *(Froggatt) and *Kladothrips waterhousei *(Mound & Crespi) comb. n. species complexes (Figure 1). The clade comprising *Kladothrips acaciae *(Moulton), *Kladothrips ellobus *(Mound), *Kladothrips maslini *(Mound, Crespi, & Kranz) and the *K. rugosus *complex, closely mirrors host exploitation (see Discussion) by the clade comprising *Kladothrips harpophyllae *(Mound, Crespi, & Kranz), *Kladothrips hamiltoni *(Mound & Crespi), *Kladothrips rodwayi *(Hardy) and the *K. waterhousei *complex. This is consistent with previous phylogenies [51], where gall-thrips species from each of these clades specialise on *Acacia cambadgei *(Baker) and *Acacia harpophylla *(Muell, Benth) and the *K. rugosus *and *K waterhousei *complexes share the same set of host species (Figures 1, 2, 3).

The monophyly of the *K. waterhousei *complex is apparently violated by *K. rodwayi*, *K. habrus*, and *K. intermedius*; similarly, *K. rugosus *is not monophyletic due to the presence of *K. maslini *and *K. nicolsoni*. The inclusion of these described species within the complexes is suspected to result from phylogenetic uncertainty, an artifact of host switching [52,53], and that the undescribed taxa in each complex are species. Genetic divergence and substantial variation in gall structure amongst each of the complex members suggests that at least some of the group comprises species. Unpublished work (MJ McLeish & BJ Crespi) indicates COI pairwise uncorrected "p" distances between a majority of species complex taxa is similar to those between described species. Although such distances should be treated cautiously when inferring species-level divergence, in one case at least the divergence has been shown to be at the species level. For example, the recent elevation of a *K. rugosus *host race inhabiting *Acacia papyrocarpa *(Benth) to species status, *K. nicolsoni *[59], is consistent with the complexes comprising putative species. The genetic distances between taxa belonging to each of the species complexes, host species-specific gall morphology, and cospeciation [52] between gall-thrips and *Acacia *is consistent with monophagy operating for these morphotypes. Regardless of the presence of some phylogenetic uncertainty, the high support for each clade that includes either the *K. rugosus *or *K. waterhousei *complexes provides the ability to assess the validity of a hypothesized increase in the rate of diversification for each of them, as the clades are expected to have co-radiated and therefore have radiated contemporaneously.

### Chronology

Penalised likelihood analysis using our Bayesian inference revealed contemporaneous radiations of two gall-thrips clades, one comprising the *K. rugosus *complex and the other *K. waterhousei*, *Kladothrips intermedius *(Bagnall), and *Kladothrips habrus *(Mound) complexes. We explored outcomes of r8s tests by assigning different combinations of fixed and constrained ages to the root and an internal node corresponding to the most recent common ancestor of the clade containing the *K. rugosus *group (Table 2), to assess the ratio (or fit) between each of our calibration priors. When the root node was fixed at 25 million years ago (mya), approximating the appearance of fossil *Acacia *pollen in Australia, the internal node corresponding to the presumed period of rapid speciation of *Plurinerves*, was dated at nearly 9 mya. This date approaches the period when arid-adapted *Acacia *are believed to have originated and therefore 25 mya was regarded as an upper limit for the age of the root node.

Fixing the age of the root node at 15 mya, and then constraining the age between 10–15 mya in two separate tests resulted in the age of the appearance of the common ancestor of the *K. rugosus *and *K. waterhousei *complexes being dated at between 3.3 and 5.3 mya (Table 2). Furthermore, we enforced a fixed divergence time of 5 mya at this node in one analysis and constrained this point between 2 mya and 5 mya in another test. The root node date estimates were 9.3 mya and 13.7 mya respectively (Table 2). Therefore, the ratio of the age of the root node and the common ancestor of the *K. rugosus *and *K. waterhousei *complexes were consistent with one another between the independent analyses.

To generate our chronogram (Figure 2), we used a gamma shape distribution value of 0.5316 generated using Modeltest 3.0 [60]. The age at the root and at the common ancestor of the *K. rugosus *complex was constrained between 10 and 15 mya and 2 and 5 mya respectively. The cross-validation analysis yielded a smoothing value of 3.981072. The chronogram indicated that the common ancestors of the *K. rugosus *and *K. waterhousei *complexes arose at approximately the same time between 3.38 and 6.05 mya. The age of the root of the phylogeny was dated at between 10.00 and 10.43 mya. Host affiliation inferred using a stochastic character mapping approach implemented in SIMMAP indicated a host switch to a *Plurinerves *ancestor (the probability of a *Plurinerves *ancestor at this node = 99.9%) at approximately 7.5 mya.

### Diversification rate

We used likelihood ratio tests to detect branch lengths that were not significantly different than zero to assess the possibility of hard molecular polytomies. The genetic data were divided into six partitions to generate Bayesian inferences. As different partitions showed branch length variation, we mapped branch lengths not significantly different from zero (at the α = 0.05 level) for 6/6 partitions, 5/6 partitions, 4/6 partitions, and no partitions onto our Bayesian consensus phylogram (Figure 1). There was a high density of internal and terminal branch lengths not significantly different from zero for 6/6, 5/6, and 4/6 partitions within the *K. rugosus *and *K. waterhousei *complexes. For the 5/6 partitions of branch lengths not significantly different from zero, the non-zero length partition was always derived from the mitochondrial DNA data (*COI *and *16S*), which might be expected given that these gene regions have higher substitution rates than the other partitions. When the data were pooled together (i.e. no partitions), branch lengths not significantly different from zero always coincided with all or a high proportion of branch lengths not significantly different from zero indicated by the partitioned analyses.

SYMMETREE performs seven slightly different alternatives of diversification rate tests, each progressively more sensitive to different nodal depth scales [61]. When testing for rate variation within a tree, significant tree asymmetry (Figure 1) was detected through the most to least sensitive test-statistics (single-tree 0.0001 < P < 0.0315; batch processing 0.000 < P < 0.000). Whole-tree analysis for the detection of tree asymmetry indicated that lineages within the gall-thrips phylogeny diversified at significantly different rates. Diversification rate shifts inferred from the best Bayesian consensus phylogeny are conditional on its accuracy and also implicitly assume full representation and/or random sampling of gall-thrips taxa. The phylogeny was lacking some gall-thrips taxa but increased the representation of known taxa from approximately 35% [51] to 90% and was assumed not to compromise the analysis.

Given significant levels of tree asymmetry, branches along which putative rate shifts occurred can be identified. The significant rate shift identified in a branch in the single-tree analysis coincides with the node of the most recent ancestor of the *Kladothrips antennatus *(Moulton) lineage (*P*Δ_1 _= 0.0363636 and *P*Δ_2 _= 0.0476392). Substantial but not significant rate shifts were identified at the most recent ancestral branch to the *Kladothrips morrisi *(Mound, Crespi, & Kranz) lineage (*P*Δ_1 _= 0.0583 and *P*Δ_2 _= 0.075) and most recent ancestral branch to the *K. rodwayi *lineage (*P*Δ_1 _= 0.066 and *P*Δ_2 _= 0.066). Of the 100 replicates in our batch processing analysis used to account for phylogenetic uncertainty, the significant diversification rate shift at the ancestor of *K. antennatus*, was consistently identified along the same branch although slight tree-topology changes occur among the replicate sample.

**Figure 4:**
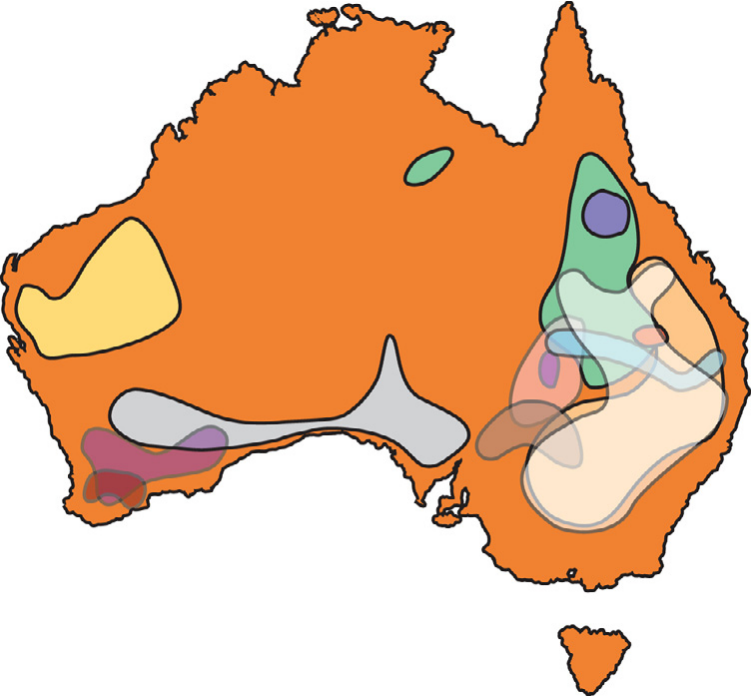
****The species ranges of *Plurinerves *hosts of the *K. rugosus *and *K. waterhousei *complexes**. **Distribution ranges' of different *Acacia *species are show by various overlapping outlined areas. Simplified from Maslin [48].

The lineage by time diversification plot (Figure 4) shows differential rates between gall-thrips that specialise on *Plurinerves *and *Juliflorae*. Known taxa that inhabit *Juliflorae *hosts not represented in the phylogeny (approximately 5% of known types) comprise a small proportion of the total known types, and therefore the general trend in diversification rates would not alter substantially. The plot indicates lineages that specialise on *Plurinerves *host species diversify at a higher rate than those on *Juliflorae *hosts and this is contemporaneous with a period of exaggerated aridification during the Quaternary period over the last 4 million yeayrs (myr).

## Discussion

On the assumption that gall-thrips and *Acacia *co-radiated, chronological conclusions indicate the common ancestor of gall-thrips arose during the commencement of long-term and widespread desertification in Australia towards the end of the Miocene epoch. The colonisation of a novel ancestral host, related to the extant *Plurinerves*, occurred before the inferred onset of climatic cycling and more rapid desert expansion over the Quaternary period. This host switch appears to have afforded greater opportunity for gall-thrips diversification on *Plurinerves *compared to species specialising on an alternative host section. Sequence divergences amongst gall-thrips specialising on *Plurinerves *suggest this group has been subject to a rapid and relatively recent diversification episode and this is consistent with processes of climatic cycling during the Quaternary period.

### Phylogenetics

Bayesian inference indicated two well-supported, well-resolved monophyletic clades: one comprising *K. ellobus*,*K. acaciae*, and the *K. rugosus *complex, and the other comprising *K. hamiltoni*, *K. harpophyllae*, and the *K. waterhousei*, *K. habrus *and *K. intermedius *complexes (Figure 1). These taxa are all affiliated with *Plurinerves *hosts; however, each of the clades includes either *K. maslini *or *K. rodwayi*, lineages that have likely undergone a host-switching event [52,53] (Figure 2). Other data [52] and phylogenetic patterns [53] indicates these gall thrips clades have diversified in parallel via some combination of cospeciation, highly restricted host-plant switching, or both processes. The less than strict form of cospeciation between these gall-thrips clades and the host species they share implies that underlying processes impacting diversification might act in tandem for both clades on highly-related *Plurinerves*. This is in contrast to lineages inhabiting more distantly-related host species.

Indications of processes driving gall-thrips diversification acting on the *K. rugosus *and *K. waterhousei *complexes might be expressed differently in *K. rodwayi *and *K. maslini *as a consequence of host switching. These two species inhabit phylogenetically distant host taxa [48,62], are mesic-zone distributed [51,63], and share secondarily acquired plesiotypic life history strategies. The inferred host switches of the *K. rodwayi *and *K. maslini *lineages might represent a shift to enemy free space [1,38]. Phylogenetic inference suggests *Koptothrips *species specialise on gall-thrips species [38] and that as a consequence might also diversify in parallel with the host thrips after the host switch [64,65] and this is consistent with the presence of *Koptothrips *parasitising *K. rodwayi *galls. Very little life history information has been collected for *K. maslini *in contrast to that for *K. rodwayi *discussed below.

The inferred host switch of an ancestor of *K. rodwayi *apparently resulted in a loss of soldiers (sociality), the retention of small gall size, small brood sizes similar to social species, and adult eclosion in the gall before dispersal [63]. The striking life history shift and presumed subsequent bottleneck effects resulting from the host switch would be expected to influence the level of sequence divergence observed in the *K. rodwayi *branch. The phylogram (Figure 1) shows a terminal branch for the *K. rodwayi *lineage similar to those of many other branches leading to described gall-thrips species. The relative level of differentiation, assuming the sampled genetic change reflects observed phenotypic change (Figure 1) might help explain why *K. rodwayi *has retained some features of closely related social species in this clade, including small brood numbers and small gall sizes (the social species include those from *K. harpophyllae *down to *K. intermedius *except for *K. rodwayi *and *K. xiphius *in Figure 1).

Life history shifts appear to be more acute subsequent to a host-switching event [52]. Dramatic life history shifts appear to have coincided with gall-thrips association with *Plurinerves *hosts. For example, gall-thrips on *Plurinerves *have evolved behaviourally and morphologically specialised defensive castes, cofounding between male and female, and adult eclosion outside of the natal gall [38]. The proliferation of gall-thrips lineages on *Plurinerves *hosts, under the assumption that the extinction rate for each group is more or less equivocal, contrasts with those on *Juliflorae *hosts and suggests that each group was subject to variation in processes affecting diversification that might be linked to host evolution. Host-related races present in the species complexes inhabiting *Plurinerves *do not occur for the *K. maslini *and *K. rodwayi *lineages on more distantly-related hosts distributed in non-arid climates. Host-plant diversity has been found to play a key role underlying the rates at which host-switching, and therefore insect diversification, can take place [66,67]. However, the opportunity for increased diversification after the successful colonisation of a novel niche need not necessarily follow, and perhaps the processes driving diversification might differ between host groups. Taken together, these patterns suggest that diversification in gall-thrips is closely linked to host evolution in addition to host affiliation.

### Chronology

We estimated absolute timing of lineage diversification and mapped host affiliation onto our Bayesian consensus tree (Figure 1). In the absence of direct evidence of gall-thrips divergence times, co-radiation between thrips and *Acacia *has been treated as an assumption and the ratio of timing between the origin and radiation of gall-thrips specialising on *Plurinerves *hosts tested. Independent analysis of each calibration point supported the timing of the alternative calibration date and a chronogram was generated using both constraints. Penalised likelihood estimates and ancestral host affiliation inferences show a host switch to a *Plurinerves *ancestor approximately 7.5 mya, before more extreme arid-zone expansion during the Quaternary period. The common ancestors of the *K. rugosus *and *K. waterhousei *complexes both occurred at approximately the same time approximately 3.6 mya. The coincidental diversification of the two complexes, each specialising on the same set of *Plurinerves *host species (Figure 3), suggest that a component of diversification opportunities for gall-thrips was a consequence of host range expansion and host speciation. Floral communities on desert margins in Australia would have been sensitive to glacial extremes and climatic cycling [21-24] and speciation in the sclerophyllous taxa was partly due to population fragmentation and lability induced by these upheavals [19,25,26].

### Diversification rate changes

Our evidence supports Hopper and Gioia's [32] prediction that phylogenetic pattern showing unresolved relationships towards the terminal branches of a tree attests to rapid increase in diversification/speciation. The high incidence of molecular polytomies and short branch lengths towards the terminal ends of the phylogeny generally occur for gall-thrips lineages that specialise on *Plurinerves *and not *Juliflorae*. The presence of hard molecular polytomies indicated that low support within the *K. rugosus *and *K. waterhousei *complexes (Figure 1) might be the outcome of rapid diversification in these lineages [68,69].

Furthermore, diversification rate variation analyses identified significant tree asymmetry at a branch believed to represent the transition from gall-thrips lineages that display a plesiotypic life history (Figure 2). The four species, *Kladothrips zygus *(Mound, Crespi, & Kranz), *Kladothrips pilbara *(Mound, Crespi, & Kranz), *Kladothrips schwarzi *(Mound, Crespi, & Kranz), and *K. antennatus *are considered to be similar in biology to ancestral gall-inducing thrips. These species display the most *primitive *morphology and gall structure, inhabit *Juliflorae *hosts, and are distributed in the most arid regions of northwestern and central Australia [38]. *Juliflorae *such as *Acacia aneura *(F. Muell ex. Benth), supports *K. antennatus *and three other centrally distributed species, *Kladothrips arotrum *(Mound), *Kladothrips sterni *(Mound, Crespi, & Kranz), and *Kladothrips tepperi *(Uzel).

The underlying causes of the diversification rate change before the origins of the *K. rugosus *and *K. waterhousei *complexes are difficult to test. The inferred rate change occurred early in the evolution of gall-thrips *Acacia *(Figure 1), and therefore might represent a footprint of central arid-zone expansion before the proposed *Plurinerves *host range expansion on the arid-zone periphery (Figure 3) resulting from climatic cycling. The central Australian desert is known to have originated as severe aridity developed during the Pliocene from approximately 7 mya [15,16,18,70]. Truly arid-zone host populations might be less evolutionarily labile than populations in semi-arid regions. Phenotypic diversity and species richness appear to have been amplified on the *Plurinerves *lineage compared to lineages supported by centrally distributed *Juliflorae *(Figure 4) over the last 4 myr assuming our chronogram is a reasonable approximation. It is possible that fragmentation featured more prominently in the *Plurinerves *during the most recent and extensive speciation event. The *Juliflorae *might have been able to maintain gene flow sufficient to arrest fragmentation effects apparently experienced by the *Plurinerves*.

## Conclusion

This work has addressed hypotheses connecting gall-thrips phylogeny with host-plant evolution and suggests that host expansion and climate change have important consequences for gall-thrips diversification. A pronounced diversification episode for gall-thrips lineages affiliated with *Plurinerves *hosts appears to have commenced between 3 and 6 mya. The inferred host-plant switch to a *Plurinerves *ancestor relatively early in the evolution of gall-thrips, preceded the onset of pronounced diversification for this lineage. In addition to the cospeciation and host switching evident for this group, *Acacia *cladogenesis would provide an expanding resource and unoccupied ecological niches to exploit. These patterns agree with the climatic cycling during the Quaternary period contriving an opportunity for some phytophagous insects, associated with a particular flora, to diversify at heightened rates with respect to others. This work suggests a component of the plant-feeding insect diversity in Australia has been passively driven by host evolution.

## Methods

### Phylogenetic analysis

The phylogenetic analyses included a great majority of known morphotypes and all described species [51]. No isolates were available for the types not included in the analyses. A comprehensive explanation of DNA extraction, PCR, and alignment protocols, and choices for substitution models is given in McLeish *et al*. [59]. Up to 1,245 bp of the *cytochrome oxidase one *(*COI*) mitochondrial gene, 444 bp of the *elongation factor one alpha *(*EF-1α*) gene, 472 bp of the *16S *(ribosomal RNA subunit) gene, and 549 bp of the *wingless *gene were amplified. Sequence data for the *K. waterhousei *complex at *EF-1α *and *wingless *gene regions was not available and absent for all analyses. Sequences were aligned using ClustalX 1.81.1sa software ([71];  accessed 24 June 2005). Insertions and deletions were removed from the sequence alignment before analyses. All nucleic acid sequence data has been lodged with GenBank under the accession numbers AY827474–AY827481, AY920988–AY921000, AY921058–AY921069, and DQ246453–DQ246516. Bayesian inferences were implemented in PAUP*b4.10 [72] and MrBayes (MrBayes 3.0b4, [73]).

To accommodate differences in substitution rate parameters and heterogeneity of base composition in our multiple gene fragment dataset, we fitted separate models to different gene partitions in the Bayesian analysis [74,75]. The sequence data used in the MrBayes analysis was divided into six partitions comprising 1st, 2nd, and 3rd codon positions of the *COI *mitochondrial data, with single partitions for each of *EF-1α*, *wingless*, and the *16S *gene fragments. We used a general time reversible (GTR) DNA substitution model with gamma distributed rates with a proportion of invariant sites. Posterior probabilities and mean branch lengths are derived from 3,000 trees taken from generations 3.5–5.0 million, sampling every 500th generation. The sampled trees were derived from post-burnin generations after the chains had reached apparent stationarity. We ran the Bayesian analysis three times to verify the repeatability of the phylogenetic outcome using the same priors as above and obtained consistent and stable posterior values. There was a 0.16% difference in the arithmetic mean of posterior probabilities between the three MrBayes runs.

The outgroup, *Rhopalothripoides *Bagnall, is the most closely related sister-genus to *Kladothrips *Froggatt [30]. The *K. rugosus *and *K. waterhousei *species complexes were chosen as ingroup taxa, as data strongly implies that these taxa have diversified recently and in parallel [52,53] and constitute the majority of known gall-thrips morphotypes remaining to be sampled. The approximation for the origin of each of these complexes is therefore expected to be contemporaneous.

### Construction of chronograms

The molecular clock hypothesis [76] of equal rates across lineages can be examined using a likelihood-ratio test. The GTR + I + G model for all gene regions in a DNA sequence data set was assumed in the analysis. The null hypothesis is that the likelihood is maximized under the constraint of equal rates across lineages (*L*_0_), that is, an enforced molecular clock model. The alternative hypothesis relaxes the clock constraint by assigning a different rate to each of the lineages; the likelihood under the alternative hypothesis is *L*_1_. The likelihood-ratio test statistic (twice the difference in the log_*e *_likelihood, -2 log_*e *_Λ; Λ = *L*_0_/*L*_1_) between the null and alternative models is approximately χ^2 ^distributed with *s *– 2 degrees of freedom. The molecular clock hypothesis was rejected at the α = 0.05 level (log_*e*_*L*_0 _= -16,925.01, log_*e*_*L*_1 _= -16,811.27, -2 log_*e *_Λ = 227.48, *P *< 0.0001).

To better accommodate unequal substitution rates among lineages, a semi-parametric penalised likelihood (PL) rate smoothing with a truncated Newton algorithm was implemented to estimate relative ages of nodes using r8s [77]. Penalised likelihood imposes constraints on rate variation combining a model allowing substitution rate variation among branches with a roughness penalty that mediates rate changes from branch to branch. A "smoothing parameter" that determines the relative contribution of each was determined by a cross-validation procedure. To generate a PL chronogram, we used MrBayes to generate a consensus phylogeny using the same priors as described above. Modeltest 3.0 [60] was used to determine the appropriate nucleotide substitution model (GTR + I + G) and gamma shape required for r8s analysis. The outgroup *Rhopalthripoides *was pruned prior to analyses using r8s. The 95% central distribution intervals for node-age estimates were generated by filtering post-burnin trees, retaining only those that had an identical topology to the consensus tree produced by our Bayesian analysis. This resulted in 258 sampled trees from the MCMC run. We used r8s to generate chronograms for each of these filtered trees. The resulting branch length estimates were used to calculate the ages of key nodes for each tree and the 2.5% right and left tails were then removed, leading to 95% central distribution limits that could be used as a measure of confidence for the point estimates [78].

### Clock calibration

In the absence of even a modest amount of evidence bearing directly on gall-thrips divergence times, fossil *Acacia *pollen and biogeographical evidence was used to calibrate the phylogeny. Therefore, chronological conclusions are only valid under the assumption that gall-thrips radiations must have coincided with host radiations. This is a reasonable proposal given that gall-thrips specialising on *Plurinerves *display a less-than-strict form of cospeciation between insect and host [52,53] and are therefore tightly affiliated with this resource. In addition, we aimed to show that the 'ratio' of the ages of two calibration points in the gall-thrips phylogeny is similar regardless of the absolute timing of these events.

Divergence events were calibrated using combinations of fixed and/or constrained maximum and minimum times at the root node and an internal node based on fossil *Acacia *pollen records and biogeographical evidence. Each of the calibration points used was individually assessed in separate analyses to compare the appropriateness of the other calibration point, and thereby determining the ratio (or fit) of calibration points. That is, the root node or an internal node was calibrated independently as a fixed age or constrained between two ages. For these tests, the root node was fixed at 25 mya and 15 mya and the internal node at 5 mya. Additionally, the root was constrained between 10–15 myr or the internal node between 2–5 myr. Constrained calibration points were presumed to be a more realistic prior because the biogeographic and fossil evidence are given in approximate terms.

Calibration points were nominated in accordance with the following evidence. The appearance of fossil *Acacia *pollen the fossil record in Australia 25 mya [79] and the distribution of the *Acacia *subgenera *Phyllodineae *hosts to which gall thrips are confined, indicate that radiations of this group occurred in Australia no more than 15 mya [24,40]. Therefore, the appearance of gall-thrips hosts presumably coincided with the appearance of an ancestor of these arid-adapted *Acacia *during the Miocene; therefore we constrained the root node between 10–15 mya. A rapid increase in fossil *Acacia *pollen co-occurs with a period of pronounced arid-zone expansion in Australia over the last 3 myr [30,79]. Evidence indicates speciation in host *Acacia *section *Plurinerves *occurred over a relatively short period as widespread aridity developed in the early Quaternary over the last 2–5 myr [15,16,53,80,81]. The common ancestor of the *K. rugosus *species complex specialising on extant *Plurinerves *species was therefore constrained to between 2–5 mya. The *K. waterhousei *species complex specialises on the same group of host species as *K. rugosus *and was therefore expected to have radiated contemporaneously.

To provide a convenient visualisation of historical host utilisation by gall-thrips, as gall-thrips are highly host specific [38], and to indicate the relative age of the transition from *Juliflorae *to the novel ancestor of host section *Plurinerves*, ancestral states were also inferred using a stochastic character mapping approach (SIMMAP: [82]). SIMMAP is a post-tree analysis program for making inferences about character evolution and implements a Bayesian method for mapping characters using stochastic substitution models [83]. To avoid potentially spurious inferences based on a single topology, character histories were sampled from the posterior distribution of 1,000 trees obtained from our MrBayes analyses. We used a data matrix of host utilization to calculate the probability of a host switch to a *Plurinerves *ancestor. Gall-thrips host species assignments for the data matrix were taken from Crespi *et al*. [53]. *Plurinerves*, *Juliflorae*, or *Phyllodinae *host affiliation was coded from 0 to 2 respectively.

### Diversification rate

To investigate diversification rate changes we used a whole-tree asymmetry approach implemented in SYMMETREE [61]. The approach is appropriate for use with trees including taxa below species-level and it is robust to polytomies. Divergences were expected to be marginal as mtDNA *COI *uncorrected "p" distances were below that between other gall-thrips species in some instances [38,52]. Detection of among-lineage diversification rate variation compares observed differences in sister-group diversity to a null distribution of sister-group diversity. Diversification rate variation within the phylogeny is reported by a range of test statistics that vary in their sensitivity to nodal depth scales [61].

Diversification rate change along a branch can also be inferred. Two different diversification rate change *P*-statistics are calculated. Each calculation considers the detection of a shift along an internal branch of a three-taxon tree in different ways [84]. The first, *P*Δ_1_, accounts for the differences between rate models and the second, *P*Δ_2_, for the possibility that the ingroup diversity might not be subject to a rate shift. The 'batch processing' option in SYMMETREE was applied using posterior probability distribution of 1,000 Bayesian trees thinned from 10,000. We used a random-resolution algorithm taxon-size sensitive (TTS) ERM branching model. The number of random resolutions was set to 100,000 whole trees. To test the reliability of the single-tree analysis and the potential phylogenetic uncertainty associated with it, we batch processed a random sub-sample of 100 trees generated from posterior probability replicates of the Bayesian analysis.

To test the hypothesis of diversification rate change further, we also used a method for detecting multifurcations, or molecular polytomies, using likelihood ratio tests [68,69], expecting a reasonable coincidence of branch lengths not significantly different from zero with regions of diversification rate change. Low-level node support of internal branches in phylogenies can be a result of insufficient data or an artifact of short periods of rapid speciation, during which little or no informative signal is trapped. We suspected a series of poorly supported branches corresponded to rapid diversification as inclusion of additional markers did not improve node support at these nodes in several instances. We used Modeltest [60] to estimate the substitution rate characteristics of each partition used in our phylogenetic analysis and used likelihood ratio tests implemented in PAUP* to detect branch lengths not significantly different from zero for separate and all partitions combined.

A lineage by time diversification plot was used to show differential rates of gall-thrips lineage diversification that specialise on either *Plurinerves *or *Juliflorae*. Lineage frequencies per time were counted using the Penalised Likelihood chronogram generated by Sanderson's r8s program [77]. The plot includes taxa most likely below that of species status where a large majority of known yet undescribed gall morphotypes [59] have been included. We assume equivocal extinction rates between the two groups and presume that several known morphotypes specialise on *Juliflorae *host species, that were not sampled, are only marginally underrepresented (see above).

## Authors' contributions

MM conceived the study. MM and TC collected the data. MM carried out the analyses, phylogenetic reconstructions, and wrote the paper. MS provided direction on available means of data analyses. All authors contributed to the general content and structure of the final manuscript.

## Figures and Tables

**Table 1:** Penalised likelihood estimates of root node and internal node using our Bayesian consensus phylogram.

Calibration Points (myr)	Smoothing value	Root age	Internal age
Root: 25	14.454	25.00	8.97
Root: 15	15.849	15.00	5.31
Root: 10–15	13.183	10.01	3.61
Internal: 5	69.183	13.66	5.00
Internal: 2–5	6.918	9.31	3.38

The internal node corresponds to the most recent common ancestor of the *K. rugosus *species complex. Ages are in units of millions of years (myr). Rate smoothing values were calculated using a cross validation method implemented in Sanderson's r8s program [83].
